# Microbiome-Metabolites Analysis Reveals Unhealthy Alterations in the Gut Microbiota but Improved Meat Quality with a High-Rice Diet Challenge in a Small Ruminant Model

**DOI:** 10.3390/ani11082306

**Published:** 2021-08-05

**Authors:** Kaijun Wang, Xiaomin Peng, Feifei Lv, Mengli Zheng, Donglei Long, Hongxiang Mao, Hongbin Si, Peihua Zhang

**Affiliations:** 1 College of Animal Science and Technology, Guangxi University, Nanning 530004, China; 2018402004@alu.gxu.edu.cn (K.W.); 1918393040@st.gxu.edu.cn (X.P.); 1918393033@st.gxu.edu.cn (F.L.); 2 Hunan Provincial Key Laboratory for Genetic Improvement of Domestic Animal, College of Animal Science and Technology, Hunan Agricultural University, Changsha 410128, China; zml199302@sina.com (M.Z.); ldl15073147518@foxmail.com (D.L.); maohongxiang@cn.wilmar-intl.com (H.M.)

**Keywords:** high concentrate diet, meat quality, microbiota, gut, goat

## Abstract

**Simple Summary:**

Effects of a high-rice dietary proportion on the meat quality, gut microbiota and metabolites in small ruminants are rarely reported. Thus, the objective of this study was to evaluate the slaughtering characteristic and meat quality, acute phase reaction proteins (APRPs) in plasma and colonic microbiota and metabolites of goats subjected to a high-rice diet. After a 35-day period, sixteen goats received a high-rice diet (HR, 90% concentrate) or a control diet (55% concentrate). In summary, the results showed that the slaughter performance and meat quality were improved in the growing goats after being fed the HR diet. However, the HR diet induced an acute phase reaction and disturbed the gut microbiota to some extent, which increases the health risk to growing goats.

**Abstract:**

Effects of a high-rice dietary proportion on the meat quality, acute phase reaction proteins (APRPs) and colonic microbiota and metabolites in goats are rarely reported. This study was designed to investigate the meat quality and metabolism in goats. Sixteen goats were equally divided into two groups and fed a control diet (Con, 55% concentrate) or a high-rice diet (HR, 90% concentrate) for five weeks. We found that the HR diet improved the slaughtering characteristic and meat quality but induced an acute phase reaction and decreased bacterial richness and diversity when compared to the control group. Furthermore, the levels of acetate, propionate and total VFA concentrations were higher in the colonic contents of the HR-fed goats than in those of the control group (*p* < 0.05). Meanwhile, the HR diet decreased the pH value, lactic acid concentration and increased the activity of amylase and lipopolysaccharide concentration in the colonic contents of goats (*p* < 0.05). The proportion of *Oscillibacter* increased while *Phocaeicola* and *Christensenellaceae_R-7_group* significantly decreased with the HR diet (*p* < 0.05). Collectively, the HR diet induced an acute phase reaction and altered the colonic bacterial community, which increases the health risk to growing goats.

## 1. Introduction

In modern ruminant production systems, to achieve maximum performance, the inclusion of plentiful amounts of concentrate into the diets is commonly practiced in the intensive feedlot management system of ruminants [1,2]. Usually, feeding large amounts of concentrate tends to advance the meat production for goats; nonetheless, feeding diets high in readily fermentable carbohydrates boosts the possibility of developing a series of metabolic diseases, such as subacute ruminal acidosis (SARA) in goats [3,4]. The presence of SARA was a major issue in terms of both productivity and animal welfare. During SARA in ruminants, many previous studies revealed gastrointestinal fermentation increased the concentrations of short-chain fatty acids (SCFA) [5,6]. Accumulation of SCFA production and digestion results in a dramatic decrease in the ruminal pH and lead to SARA, which could depress the dry matter intake and fiber absorption, as well as alter meat quality [7]. It has been proved that SARA induced by a high concentrate diet might cause an acute phase reaction with an increase in acute phase reaction proteins (APRPs) in blood [8,9]. The acute phase reaction helps the body to restore its destroyed physiological homeostasis [10]. Therefore, it is necessary to establish a high grain diet model to study the effects of acute phase response on goats.

In fact, because corn is generally lacking in the traditional rice region of southern Asia, rice is used as an alternative feed applied in the goat model. Diet is a key regulator to regulate the composition and establishment of intestinal microflora [11]; nonetheless, feeding a high concentrate diet is widely adopted to provide adequate protein and energy supply for meeting higher performance needs at the finishing stage of ruminants [12]. Increases in dietary concentrate can alter the rumen and gut bacterial composition, but these factors range extremely among ruminants [13,14,15]. Previous studies have reported that the intestinal bacterial community plays a key role in regulating oxidative stress, the immune system [16], metabolic disease [17] and inflammation [18]. Notwithstanding, we hear nothing of what happens to the meat quality of goats nor of the microbial composition of the colon caused by an HR diet. Such studies are essential to strengthen the comprehension of the relationship between HR diet-induced SARA and the intestinal bacterial community of ruminants, as this may lay the foundation for the development of strategies to prevent SARA.

Sixteen growing goats were selected as the experimental model in the present study. The application of Illumina MiSeq sequencing methods would help to explain the underlying mechanism of the comprehensive variation in colonic microbiota caused by feeding animals an HR diet. We hypothesized that SARA induced by an HR diet might cause variations in the meat quality, acute phase reaction of blood, colonic fermentation and metabolic activities, and further change the microorganisms in growing goats. Consequently, the objective of this study was to determine the metabolic changes in goats after an acute phase protein reaction, and investigate the changes in goat meat quality, colonic microbiota, fermentation products and biochemical parameters in goats during HR feeding. Additionally, the relationship between alterations in the microbial composition and physiological parameters was also evaluated in the colonic contents of goats.

## 2. Materials and Methods

### 2.1. Animals, Diets and Management

In this study, we randomly selected sixteen Liuyang Black goats (6 months old) as experimental models, with an average live weight of 15.3 ± 1.67 kg; these were divided into two groups, fed the control diet (concentrate/hay was 55:45) and high-rice diet (HR, concentrate/hay was 90:10). The ingredients and nutrient levels of the diets are given in Supplementary Material Table S1. The diet composition included rice straw, the most commonly used forage in South China, selected as the roughage for goats, whereas rice with the shell was the main concentrate, supplemented with soybean meal and wheat bran. The purpose was to explore the effect of rice with the shell as feed on goats. The experiment duration consisted of 35 days, with 7 days for diet adaptation. Diets were equally offered at approximately 08:00 and 18:00 h, respectively. All goats were fed in separate cages.

### 2.2. Sampling and Collection

Plasma samples were collected in tubes with heparin sodium from the jugular vein of goats on Days 34 and 35. The samples were separated by centrifugation at 1000× *g* for 15 min, and stored at −20 °C until analysis. At the end of the experiment on Day 35, six animals were eventually selected from each group and slaughtered at random. Slaughtering performance and meat quality were measured (longissimus dorsi muscle was taken for measurement). Colonic contents were collected and then stored at −80 °C for genomic DNA isolation, analysis of metabolic indicators and SCFA. Colonic segments from each goat were collected for observation of intestinal morphology. The colonic tissues were dehydrated, embedded, and cross sections of the segments were cut approximately 5 mm thick using a microtome and stained with hematoxylin and eosin. Representative photographs of intestinal morphology of the colon were collected using a light microscope with a computer-assisted morphometric system. Feces were collected from goats twice daily before feeding and lasted for 7 days. For feces samples, further subsamples were acidified with 10% H_2_SO_4_ and dried at 65 °C for the observation.

### 2.3. Slaughtering Performance and Meat Quality Measurement

Carcass weight: The weight of the whole body after resting, slaughtering and bloodletting, with the fur, head, viscera and parts below the knee and pubic joints of the forelimbs and hindlimbs removed. Dressing percentage: Percentage of carcass weight after slaughter to live weight before slaughter. Loin eye area: the transverse area of the longissimus dorsi between the 12th and 13th ribs. The cross section of the loin eye muscle was drawn with sulfuric acid drawing paper and the area of the loin eye muscle was calculated as follows:

Loin eye area (cm^2^) = loin eye muscle height × loin eye muscle width × 0.7

pH measurement of meat: A pH meter (Russell CD700, Russell pH Limited, Germany) was used to measure the pH 45 min and pH 24 h of the longissimus dorsi muscle (vertebrae 12–13) 45 min and 24 h after slaughter, respectively. The pH of the upper, middle and lower parts of the longissimus dorsi muscle were measured, and the average value was determined for each sample. Meat-color measurement: Color measurements of lightness (L*), redness (a*) and yellowness (b*) were carried out on the surface of the longissimus muscle between the 12th and 13th ribs. The measurement was undertaken at three random locations of each sample using a Konica Minolta Chroma Meter (CR410, Konica Minolta Sensing, Tokyo, Japan), and the average was taken for each sample.

### 2.4. Acute Phase Proteins Levels in the Plasma Measurement

The acute phase reaction proteins (APRPs) of plasma, including SAA (serum amyloid A protein), HP (haptoglobin), LBP (lipopolysaccharide binding protein), CER (ceruloplasmin) and CRP (C-reactive protein), was detected using the corresponding ELISA kit (Jiangsu Yutong Biological Technology Co., Ltd., Yancheng, China).

### 2.5. DNA Extraction and PCR Amplification

The DNA extraction of colonic contents were consistent with that reported in the previous article [19]. It was performed according to the instructions of a DNA Stool Mini Kit (Qiagen, Hilden, Germany) [20]. The bacterial universal V3-V4 region of the 16S rRNA gene was amplified according to PCR barcoded primers 338F (5′-ACTCCTACGGGAGGCAGCAG-3′) and 806R (5′-GGACTACHVGGGTWTCTAAT- 3′). PCR was performed in a total of 20 μL volume, containing 1 × FastPfu Buffer, 250 μM dNTP, 0.1 μM each of the primer, 1 U FastPfu Polymerase (Beijing TransGen Biotech, Beijing, China) and 10 ng template DNA. PCR was performed at 95 °C for 2 min, followed by 30 cycles of 95 °C for 30 s, annealing (55 °C, 30 s), elongation (72 °C, 30 s) and a final extension at 72 °C for 5 min. Afterward, the amplification products were verified through 2% agarose gel electrophoresis. Purified amplicons were pooled in equimolar and paired-end sequenced (2 × 300) on an Illumina MiSeq platform (Allwegene, Beijing, China) according to the standard protocols.

### 2.6. Bacterial Data Processing

The raw data were filtered and analyzed using QIIME (version 1.17). Sequences were clustered into operational taxonomic units (OTUs) with 97% similarity using UPARSE (version 7.1, http://drive5.com/uparse/, 2018), and chimeric sequences were removed using UCHIME. The rarefaction analysis based on Mothur v.1.21.1 was conducted to reveal the alpha diversity (Good’s coverage, Chao 1, Shannon and Simpson). Principle Component Analysis (PCA) was conducted to identify the differences between the control and high rice group.

### 2.7. Metabolites of Fermentation and Biochemical Parameters

The SCFA was analyzed from chromatograph peak areas using gas chromatography (Agilent 7890A, Agilent, Wilmington, DE, USA), according to the method described in our previous work [19]. Meanwhile, the pH values of the colonic chyme fluid were determined using a pH meter (Model PHS-3C, Shanghai Precision Science Instrument Co., Ltd., Shanghai, China). The colonic biochemical components including lactate (LACT), lactate dehydrogenase (LDH), aminotransferase (AST), alanine aminotransferase (ALT), aspartate alkaline phosphatase (ALP), amylase (AMY) and lipopolysaccharide (LPS) in the colon was detected using the corresponding ELISA kit (Jiangsu Yutong Biological Technology Co., Ltd., Yancheng, China).

### 2.8. Statistical Analysis

All statistical analyses were conducted with SPSS 19.0 (SPSS Inc., Chicago, IL, USA, 2009). First, the data were evaluated through the Shapiro–Wilk test to check whether the distribution of the variables exhibited a normal distribution. Then, the variables that showed a normal distribution and a non-normal distribution were analyzed by the independent sample t test and the Kruskal–Wallis test, respectively. Statistical significance was set at *p* < 0.05 and tendencies at 0.05 ≤ *p* ≤ 0.10.

## 3. Results

### 3.1. Slaughter Characteristic and Meat Quality of Goat

**Figure 1:**
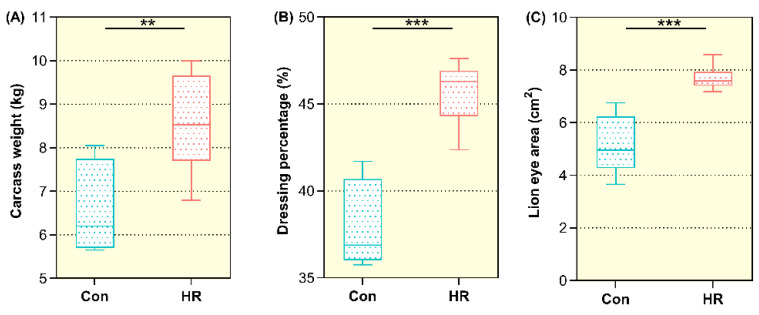
The slaughtering characteristic of growing goats fed the control and high-rice diet. Con, control diet; HR, high-rice diet. (**A**) The carcass weight of goats between control and high-rice diet. (**B**) The dressing percentage of goats between control and high-rice diet. (**C**) The loin eye area of goats between control and high-rice diet. “**” means that the difference between the two groups is significant (*p* < 0.05), and “***” means that the difference between the two groups is extremely different (*p* < 0.01).

To assess the effect of feeding goats an HR diet on carcass weight, the carcass weight of all goats was measured after removal of viscera. The results presented in Figure 1A showed that the mean carcass weight of the HR group was significantly higher than that of the control group (6.62 ± 1.07 vs. 8.57 ± 1.15, *p* < 0.05). The data also showed that the dressing percentage of the goats was improved notably by the HR diet (*p* < 0.01) (Figure 1B). The HR diet also raised the loin eye area of the goats remarkably (*p* = 0.001) (Figure 1C).

Meat pH values influence the shelf life, flavor and aroma of stored meat. In the present study, to assess whether HR diet affected meat pH, we measured the pH of meat steak from longissimus dorsi muscle at 45 min and 24 h after slaughter. The results shown in Table 1 demonstrated that there was no significant difference in pH 45 min (*p* = 0.251) and pH 24 h (*p* = 0.110) among the treatment groups. Meat color is an important attribute that influences consumer preference. The data in Table 1 revealed that there were no significant differences in redness of longissimus dorsi at 45 min and 24 h after slaughter (*p* > 0.05). In addition, 45 min after slaughter, the yellowness of the meat was significantly reduced by feeding a high-rice diet (*p* = 0.006), but, 24 h afterwards, it only showed a tendency to decrease (8.51 ± 1.01 vs. 7.28 ± 1.14, *p* = 0.076). However, the HR diet reduced significantly the lightness of the meat at 45 min (*p* = 0.001) and 24 h (*p* < 0.001) after slaughter. The lightness of the meat among the experimental groups indicates that a high-rice diet is a factor influencing meat color in goats.

### 3.2. Acute Phase Reaction Proteins in Blood

**Figure 2:**
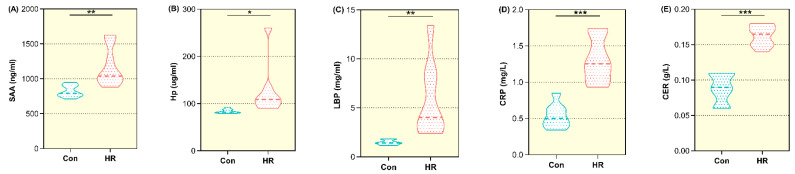
The acute phase reaction proteins concentrations in plasma of growing goats fed the control and high-rice diet. Con, control diet; HR, high-rice diet; (**A**–**E**) The serum amyloid A protein (SAA), haptoglobin (HP), lipopolysaccharide binding protein (LBP), C-reactive protein (CRP) and ceruloplasmin (CER) between the two groups in the plasma of goat. “*” means that the difference between the two groups has a significant trend (0.05 < *p* < 0.01), “**” means that the difference between the two groups is significant (*p* < 0.05), and “***” means that the difference between the two groups is extremely different (*p* < 0.01).

As shown in Figure 2, the acute phase reaction proteins (APRPs) in blood were mainly composed of SAA, LBP, Hp, CRP and CER. Compared with the control group, the plasma SAA concentration in the HR group was increased significantly (*p* = 0.010) (Figure 2A), and the LBP concentration also was raised dramatically in plasma by feeding an HR diet to goats (*p* = 0.015) (Figure 2C). Plasma concentration of Hp increased in the HR group but was not significantly different compared to the control group (83.9 ± 4.70 vs. 126 ± 56.1, *p* = 0.052). Results for plasma CRP was significantly boosted for the HR group compared to the control group (*p* < 0.001) (Figure 2D). The concentration of CER in plasma increased significantly when the dietary concentrate ratio increased from 55% to 90% (0.09 ± 0.02 vs. 0.16 ± 0.01; *p* < 0.001) (Figure 2E).

### 3.3. Colonic Bacteria Richness and Diversity by Alpha-Diversity Analysis

**Figure 3:**
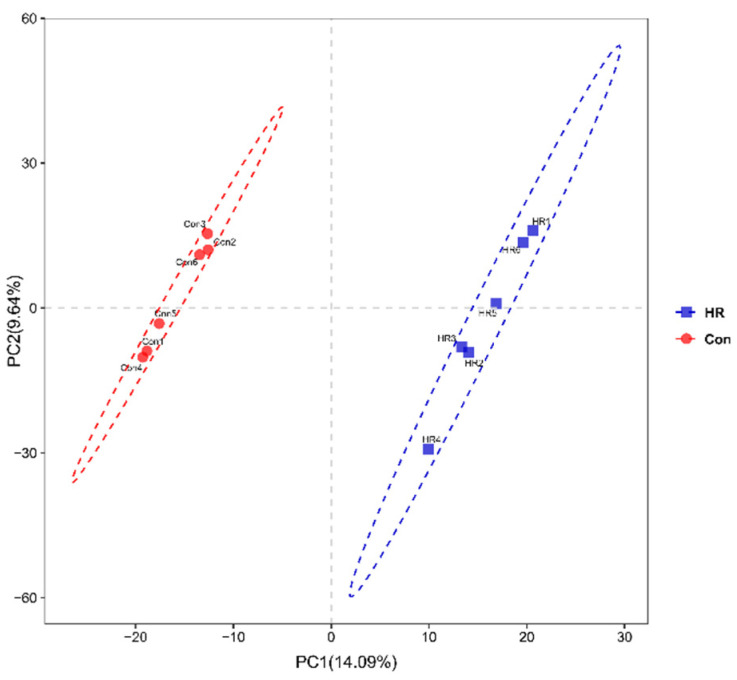
Principal component analysis (PCA) of the colonic bacterial community.

**Figure 4:**
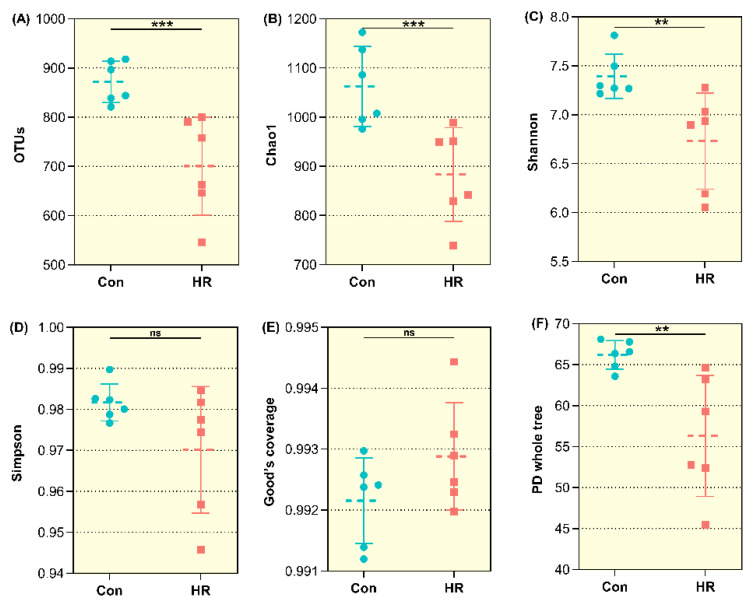
Alpha diversity indices of the colonic bacterial communities in growing goats. Con, control diet; HR, high-rice diet. (**A**) The operational taxonomic units (OTUs) between the two groups in the colon of goat. (**B**) The bacterial richness in the colon estimated by the Chao 1 value. (**C**,**D**) The bacterial diversity in the colon estimated by the Shannon and Simpson index. (**E**) The sequencing depth between the two groups in the colon of goat. (**F**) The bacterial diversity in the colon estimated by the PD whole tree. “ns” means there is no difference between the two groups (*p* > 0.1), “**” means that the difference between the two groups is significant (*p* < 0.05), and “***” means that the difference between the two groups is extremely different (*p* < 0.01).

The Good’s coverage, OTUs and statistical estimates of species richness (Chao 1) and diversity (Shannon, Simpson) between two groups at a genetic distance of 3% are presented in Figure 3 and Figure 4, respectively. All samples reached a stable plateau based on rarefaction curve analysis (Figure S1), suggesting the sampling was enough for most of the bacterial communities. The OTU community comparisons by PCA showed that samples from the colon in the Con group were well separated from those in the HR group (Figure 3). In terms of alpha bacterial diversity (Figure 4), no difference was observed across treatments for the Good’s coverage (Figure 4E), indicating that the sequencing depth was comparable across treatments (*p* > 0.05). The OTUs were markedly higher in the control group than that in the HR group (*p* = 0.006) (Figure 4A). Meanwhile, in the colonic contents, when the diet administered to goats was switched from a 55% concentrate diet to 90% concentrate diet, HR diet notably decreased the Chao 1 (*p* = 0.006), Shannon index (*p* = 0.020) and PD whole tree (*p* = 0.021) (Figure 4B,C,F). Hence, the HR diet decreased the colonic bacteria richness and diversity.

### 3.4. Intestinal Bacterial Community Structure

**Figure 5:**
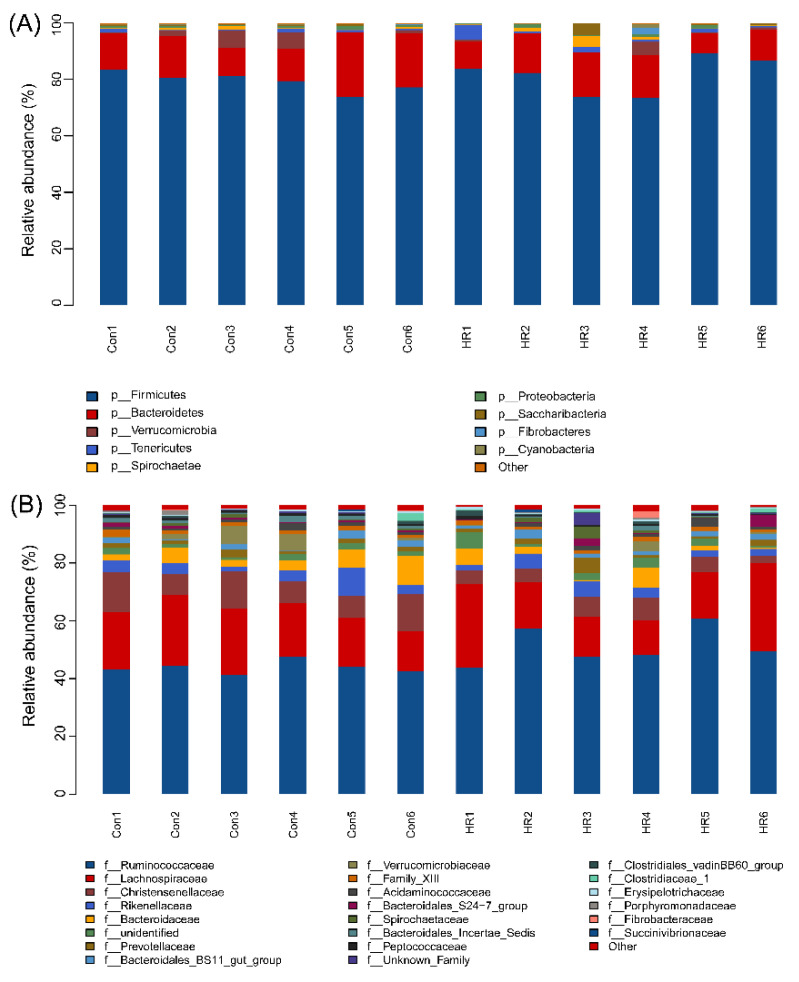
The intestinal bacterial community structure of the goats. (**A**) Distribution of colonic bacteria at the phylum level between dietary treatments in goats. (**B**) Distribution of the colonic bacteria at the family level between dietary treatments in goats. Con1–Con6 and HR1–HR6 are the colonic samples of goats fed with 55% or 90% concentrate, respectively.

Firmicutes, Bacteroidetes and Verrucomicrobia were dominant phyla in the colon of goats, accounting for more than 90% of the total colonic bacterial community (Figure 5A). Firmicutes and Bacteroidetes accounted for a relative abundance of 73.48–89.31% and 7.12–22.77%, respectively, followed by Verrucomicrobia at 0.33–13.20%. When the diet’s concentrate proportion increased from 55% to 90%, the abundance of the phylum was not significant different between the control and HR group (*p* > 0.05). At the family level, sixty-four families were identified, with the abundance of twenty-two families among them being ≥1% (Figure 5B). The Firmicutes of the colonic bacteria community were mainly composed of *Christensenellaceae*, *Ruminococcaceae* and *Lachnospiraceae*; the *Bacteroidetes* consisted of *Bacteroidaceae*, *Bacteroidales_Incertae_Sedis*, *Prevotellaceae* and *Rikenellaceae*; and *Spirochaetae* consisted of *Spirochaetaceae*. There are three families with significant differences. The proportion of *Christensenellaceae* (*p* = 0.012) and *Bacteroidales_Incertae_Sedis* (*p* = 0.042) dropped dramatically in the HR group. However, the HR diet significantly increased the abundance of *Ruminococcaceae* (*p* = 0.037).

**Figure 6:**
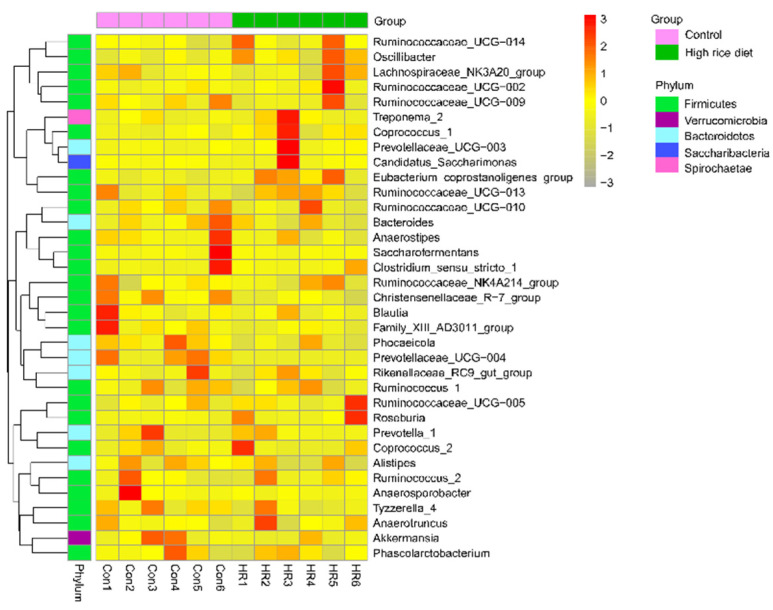
Heatmap based on the hierarchical clustering solution at the genus level (Bray–Curtis distance metric and complete clustering method) for goats fed a control diet or a high-rice diet, shown across columns.

**Figure 7:**
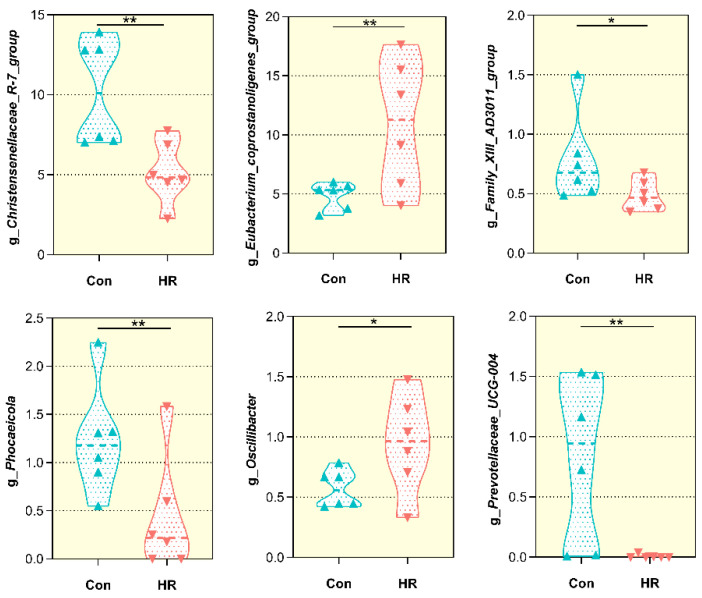
Relative abundance of microbial genera (percentage) in goat colons that were significantly affected by dietary treatment. Con, control diet; HR, high-rice diet. “*” means that the difference between the two groups has a significant trend (0.05 < *p* < 0.01), “**” means that the difference between the two groups is significant (*p* < 0.05).

Within the bacterial population, the top 35 genera were identified across all samples (Figure 6); the genus *Ruminococcaceae_UCG-005* was the most abundant genera, accounting for 12.47–30.69%, followed by *Eubacterium_coprostanoligenes_group*, *Christensenellaceae_R-7_group*, *Ruminococcaceae_UCG-010* and *Bacteroides*, which are predominant genera of colon in the control and HR group. Among the genera, the percentage abundance of the *Eubacterium_coprostanoligenes_group* (4.89 ± 1.14 vs. 11.0 ± 5.45; *p* = 0.041) was greater in the colon of the HR group when compared to that in the control group (Figure 7), and *Oscillibacter* tended to be greater after HR diet (0.57 ± 0.15 vs. 0.95 ± 0.40; *p* = 0.060). In contrast, the relative abundance of *Phocaeicola* (1.23 ± 0.58 vs. 0.44 ± 0.60; *p* = 0.042), *Christensenellaceae_R-7_group* (10.2 ± 3.31 vs. 5.18 ± 1.93; *p* = 0.013) and *Prevotellaceae_UCG−004* (0.83 ± 0.70 vs. 0.01 ± 0.02; *p* = 0.034) were markedly decreased in the colon of the HR group than that in the control group, and *Family_XIII_AD3011_group* tended to decrease after HR diet (0.79 ± 0.37 vs. 0.49 ± 0.13; *p* = 0.096).

### 3.5. Fermentation and Biochemical Parameters in the Colonic Contents

**Figure 8:**
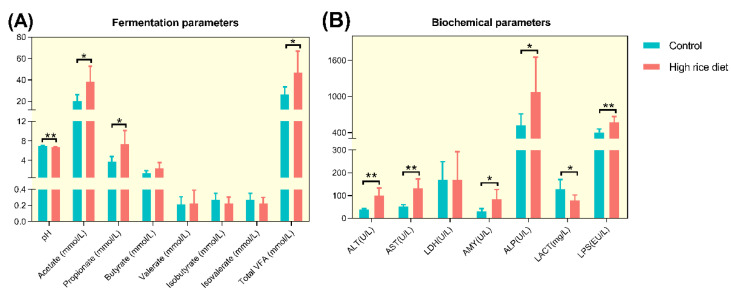
(**A**) Comparison of colonic pH and short-chain fatty acids (SCFAs) among dietary treatments in goats. (**B**) Comparison of the colonic biochemical parameters between dietary treatments in goats. “*” means that the difference between the two groups is significant (*p* < 0.05), and “**” means that the difference between the two groups is extremely different (*p* < 0.01).

As shown in Figure 8A, the pH level was decreased significantly in the colonic content after the HR diet (6.99 ± 0.09 vs. 6.66 ± 0.14; *p* = 0.001), and the concentration of acetate (20.4 ± 5.89 vs. 38.0 ± 14.8; *p* = 0.022) and propionate (3.75 ± 0.99 vs. 7.39 ± 2.72; *p* = 0.020) in the colonic content increased significantly when the dietary concentrate ratio increased from 55% to 90%, and the concentration of butyrate, isobutyrate, valerate and isovalerate had no significant difference between the control and HR group in the colon (*p >* 0.1). Besides, the dietary treatment of HR significantly increased the concentration of total VFA (26.2 ± 7.32 vs. 46.7 ± 20.2; *p* = 0.041).

**Figure 9:**
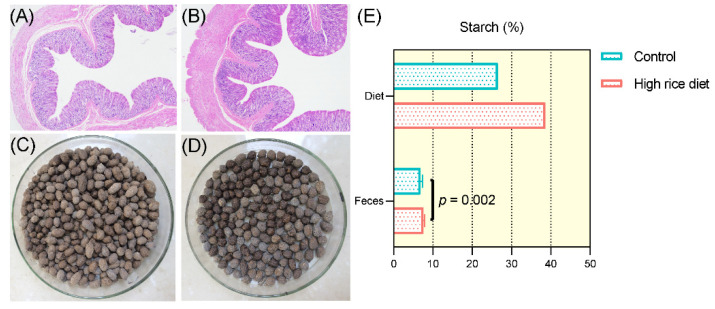
Intestinal morphology and fecal observation of goats. Light microscopy cross-section of the colon tissue from a representative control diet-fed goat (**A**) or HR diet-fed goat (**B**). Fecal photos of goats fed a control diet (**C**) or HR diet (**D**). (**E**) Starch content in goats after applying the two diets and in the feces after being fed the control diet or HR diet.

Data of the biochemical parameters between the control and HR group in the colon is displayed in Figure 9B, and compared with the control group, the HR group contained higher LPS concentrations in the colonic contents (0.40 ± 0.06 vs. 0.57 ± 0.10; *p* = 0.006). The concentration of LACT activity decreased significantly when the dietary concentrate ratio increased from 55% to 90% (0.14 ± 0.05 vs. 0.09 ± 0.03; *p* = 0.033), while there was no difference in the activity of LDH in the colonic contents (167 ± 80.5 vs. 169 ± 124; *p* > 0.1). Compared to those fed the control diet, in the colon, the HR-fed goats had higher ALT, AST, ALP and AMY activities (*p* < 0.05).

### 3.6. Correlation between Bacterial Community and SCFA and Biochemical Indices

To further examine the potential role of specific bacterial genera in the fermentation and physiological capacity in the colon of the host, Spearman’s correlation analysis was conducted between genus proportion and colonic fermentation and physiology parameters. As indicated in Table 2, the genera of Phocaeicola (r = 0.71, *p* = 0.010), Christensenellaceae_R-7_group (r = 0.61, *p* = 0.036) and Prevotellaceae_UCG-004 (r = 0.71, *p* = 0.009) were positively correlated with the pH; however, Eubacterium_coprostanoligenes_group was negatively correlated with the pH in the colon (r = 0.71, *p* = 0.024). The abundance of Christensenellaceae_R-7_group, Family_XIII_AD3011_group and Prevotellaceae_UCG-004 was negatively correlated with acetate, propionate and total VFA in the colon (r < −0.55, *p* < 0.05). The abundance of Oscillibacter was positively correlated with ALT (r = 0.63, *p* = 0.028), AST (r = 0.70, *p* = 0.012) and AMY (r = 0.72, *p* = 0.008). In addition, the abundance of four taxa (Christensenellaceae_R-7_group, Family_XIII_AD3011_group, Phocaeicola and Prevotellaceae_UCG-004) were negatively correlated with the activity of AST and AMY (r < −0.55, *p <* 0.05) in the colon. Meanwhile, only Phocaeicola was positively correlated with LACT (r = 0.71, *p* = 0.010), and Eubacterium_coprostanoligenes_group was negatively correlated to it (r = −0.63, *p* = 0.027).

### 3.7. Intestinal Morphology and Fecal Observation of Goats

Representative light micrographs of the cross sections of the colonic morphology of the control diet-fed and HR diet-fed goats are shown in Figure 9A,B. Effects of the HR diet on the colonic tissue morphology of the goats showed that the orifices of the crypts were circular in outline and the intercryptal surface was partially covered by an irregular layer of mucus, and the microvillus clusters were clear and well organized (Figure 9B). Fecal photos of goats fed the control or HR diet are shown in Figure 9C,D. The feces of the goats in the HR group had a deeper color than the control group after adding sulfuric acid and dried. As shown in Figure 9E, the content of starch in the diet of the HR group was higher than that of the control group (38.57 vs. 26.57). Furthermore, the starch content of the feces was increased obviously by feeding the HR diet (7.55 ± 0.27) compared to the control diet (6.86 ± 0.44) (*p* < 0.01).

## 4. Discussion

Slaughter performance can reflect the growth of animals. Dressing percentage and carcass weight are directly related to the meat productivity of animals and affect the economic benefits. Carcass dressing percentage was similar in the two groups and within the previously published range of 37–55% [21]. This study showed that an HR diet can significantly increase the carcass weight and dressing percentage of goats, these two indicators of goats showed an increase with the increase in concentrate level, which was consistent with the findings of Majdoub-Mathlouthi et al. [22] and Papi et al. [23]. Sen et al. [24] believed that the size of the loin eye area was positively correlated with body weight before slaughter. In this study, the changeable rule of the body weight before slaughter and the loin eye area data of goat confirmed the conjecture of Sen et al. The loin eye area was also positively correlated with the carcass weight. This may be due to the fact that the high-rice diet contained more carbohydrates, which could be absorbed by goats after carbohydrate decomposition and promoted the growth of goats, resulting in the simultaneous increase of the loin eye area and carcass weight.

pH is an important indicator that could affect meat quality. It not only directly affected the palatability, tenderness and cooking loss, but also had a significant correlation with meat moisture and meat color [25]. After the animal was slaughtered, the blood circulation stopped, and the muscle cells changed from aerobic respiration to anaerobic respiration. A large amount of muscle glycogen was mobilized to produce lactic acid, which led to a decrease in pH, and a decrease in muscle pH was also one of the important indicators for evaluating meat quality [26]. The study of Priolo et al. [27] showed that the muscle pH of sheep at 24 h after slaughter decreased with an increase in the dietary concentrate level. Mushi et al. [28] considered that the change in muscle pH was closely related to the metabolism of glycogen in muscle cells. The storage of muscle glycogen would be low when the low-energy diet fed to lambs, and the amount of hydrogen ion (H^+^) and LACT produced by anaerobic digestion was less, thus increasing the muscle pH [29]. In our study, it was also confirmed that the pH value of the goat muscles after slaughter decreased with an increase in the dietary concentrate level. However, the HR diet did not have a significant effect on the post-slaughter pH value of meat.

Meat color was one of the indicators to evaluate the quality of meat. It was often expressed in terms of redness, yellowness and lightness, which could affect consumers’ desire to buy. The higher lightness value represented the brighter the surface of the muscle. The change of redness value was related to the concentration of pigments in the muscle, such as myoglobin and hemoglobin, which showed red, and the increase of redness value within a certain range indicated the meat was bright red. A lower yellowness value indicated less pale meat. A study proved that consumers had higher acceptance of meat with pH at 5.6~6.4 [22]. As can be seen from the results, the low-concentrate diet resulted in a muscle pH higher than 6.4, which may reduce consumers’ purchase desire. In addition, Insausti et al. [30] and Luciano et al. [31] had reported that there was a significant positive correlation between the lightness and yellowness values and there was a certain inverse correlation with body weight before slaughter [32]. This conclusion was supported by the pattern of variation between lightness and yellowness values and body weight before slaughter in the present study. Meat color was the most intuitive indicator of meat quality. When the lightness and yellowness values are lower, the redness value is higher, indicating the better meat color [33]. According to our results, the meat color of goats fed the high-rice diet was better than that of the control group, which indicated that increasing the concentrate was beneficial to the meat color.

The APRP of tissue injuries or disorders is involved in the restoration of physiological homeostasis [34]. Both Hp and SAA had major roles in the innate immune system by opsonizing pathogens and removing the potential toxic substances [35]. A study demonstrated that the Hp but not SAA concentration was remarkably elevated by a high concentrate diet [36]. On the contrary, the grain-induced SARA increased the SAA concentration, but did not affect the HP concentration in cows [37]. These results may be due to the difference in animal species between goat and cow. There was consistent with present study that an increase in the blood LBP concentration in the SARA model indicated that LPS translocated from the digestive tract into the blood to elicit a systemic inflammatory response [9]. The liver is an important immune organ. The immune cells would be activated to synthesize or secrete cytokines when foreign antigens in the digestive tract encounter the immune system [38,39]. Thus, an increased entry of LPS from the colon into the liver via the portal vein probably stimulated liver macrophages to release cytokines that subsequently enhanced the secretion of APRPs from hepatocytes during feeding the HR diet. CRP and CER also were the markers of inflammation and raised together by the HR diet. This study proved the SAA, LBP, CRP and CER concentrations in blood were notably increased, suggesting that feeding the HR diet to goats caused a systemic inflammatory response. In conclusion, the concentration of APRPs in blood might be a potential indicator of SARA in goats.

A previous study reported feeding a high concentrate diet can reduce the diversity of the intestinal microbiota of cattle [40], our data also showed a decline in the bacterial richness and diversity of the microbiome in the colon after being fed an HR diet. Both the Chao 1 estimate and Shannon indices in the HR group were observed to be decreased, and a separation between colonic samples of the two groups could be best observed from PC1 and PC2. The results of the PCA further revealed the difference in bacterial diversity composition between the control and HR group. Changes in the content of the dietary nutrient between the control and the HR group might have resulted in the variations in colonic microbial response. The findings of Wetzels et al. may explain this situation [41]: the lower colonic pH level may decrease the richness of some colonic bacteria due to their sensitivity to the low pH in this study, while increasing the abundance of some low pH-tolerant colonic bacteria. Hence, feeding goats the HR diet tended to decrease the gut bacterial diversity, and the HR diet caused a negative effect on the intestinal ecosystem. It has been suggested that bacterial communities high in species richness and evenness make more efficient use of their resources, because species are different in function and specialization, and they use the part of the digestive tract that limit substrate resources [42]. So, the reduction in species richness and diversity by HR diet, suggest that the colon bacteria were transformed into a less functional and desirable state.

In accordance with previous studies that reported the intestinal bacterial communities of ruminants [19,43], our results revealed Firmicutes were the most dominant phylum and contained the most bacteria—over 73% of the total sequences from the colon chyme of goats. It is known for a fermentative metabolism and degradation of carbon sources and amino acid [44]. Hence, the abundant Firmicutes in the colon chyme bacteria make known that Firmicutes had a role in the utilization of carbohydrates and amino acids. In the current study, the Bacteroides population was no difference between the goats in the two groups, so this phenomenon could not explain that HR diet’s increased LPS level in the colon. Jenkins et al. [45] researched that species belonging to the *Christensenellaceae* family were common in the rumen and that these species played a key role in maintaining gastrointestinal structure and function. The decrease in the abundance of *Christensenellaceae* in this study indicated that the tissue of the colon will be damaged. We have not observed the damage of the colonic epithelium at present. It may be that the time of feeding the HR diet was not long enough. *Ruminococcus* is the most dominant genus found in the large intestine of healthy sheep [46] and plays a major role in degrading starch [47]. A great number of *Ruminococcaceae* were found in the HR group; a possible explanation is that the HR diet provided suitable nutrient conditions for *Ruminococcaceae* to grow, while the shift in nutrient sources in the control group resulted in its decrease [48]. This shift may be attributed to the toxic effect of the linearly increased SCFA concentrations in the HR group. Some previous studies have revealed that organic acids (acetate or lactate) can freely diffuse into bacterial cells through bacterial membrane and dissociate inside the bacterial cell, which may reduce the intracellular pH and damage the internal cell [49,50,51]. Liu et al. [52] studied that a high concentrate diet increased the populations of *Prevotella*, *Turicibacter*, *Treponema* and *Clostridium* in the hindgut of goats. There was some variation in the abundance of *Prevotellaceae*: the present study revealed that no difference in *Prevotella_1* and *Prevotellaceae_UCG-003* in the two groups, but *Prevotellaceae_UCG-004* showed a marked drop in the colon of goats after the HR diet. *Turicibacter* in the gut may be a cause of infection or other deleterious effect in the digestive tract [53]; however, the *Turicibacter* population was less than 1% in the colonic chyme of goats in the current study. Previous research indicated that some *Clostridium* spp. are pathogenic factors of intestinal disease [54] and it could alter the intestinal barrier in animals [55]. There were no differences in the abundance of *Clostridium_sensu_stricto_1* and *Treponema_2* in the two treatments. These results partly explain why the colonic epithelium was not damaged in this study.

In our previous study, feeding a high concentrate diet to goats increased the concentration of SCFA and reduced the pH value in the small intestine [15]. The diet containing plenty of carbohydrates also increased the microbial fermentation in the hindgut, which may cause an increase in the amount of starch flowing into the hindgut from the rumen and small intestine [5], and the characteristic of high concentrations of SCFA in the contents [3,6]. Studies on the influence of a high concentrate diet on SCFA in the rumen and hindgut of dairy cows and goats have been widely explored [4,5]. However, no information is available on changes in the SCFA in the colon during an HR diet. The major impact of the HR diet on the activity of the colonic microbial community in goats can be found in the production of fermentation products, mainly consisting of acetate, propionate and butyrate. As has been mentioned in this study, the HR diet decreased the pH of the colon and increased the colonic total VFA concentration. Thus, we inferred that the higher SCFA in the colon of HR-fed goats may be due to the fact that a lot of starch was fermented in the colon.

Many studies have shown that feeding ruminants with a high concentrate diet will increase the concentration of LPS in the gastrointestinal tract and causes remarkable alterations in gastrointestinal bacterial structure and diversity, in turn causing damage to the epithelium of gastrointestinal tissue [56,57]. In the present study, significantly negative correlations were observed between the concentration of free LPS in the colonic chyme and the proportion of Gram-negative bacteria, including the taxa *Phocaeicola* and *Prevotellaceae_UCG-004*. Moreover, the abundance of the two taxa was much lower in the HR group than in the control group. Our research proved that HR feeding could lead to the death and lysis of some Gram-negative bacteria, thereby increasing the concentration of free LPS in the colon. The elevated level of enzymes (ALT, AST and ALP) indicated some damage or changes in membrane permeability [58]. Therefore, the long-term feeding of an HR diet may damage the intestinal epithelium of goats. However, no pathological changes in colonic epithelium were found in our current study, which may be due to the short feeding time. The LDH was released during tissue damage and as a marker of common injury and disease [59]. Our data display that there was no difference in the activity of LDH in the two treatments. So, the result of no pathological change in colon tissue was consistent with the LDH activity not being affected by the HR diet. The HR diet increased the activity of AMY in the colon, similar to previous results where a greater starch intake increased the AMY activity in the pancreas and small intestine contents [60,61]. We also found that *Christensenellaceae_R-7_group*, *Family_XIII_AD3011_group* and *Phocaeicola* were negatively correlated with the activity of AMY in the colon. These results may indicate that the higher AMY activity may be partly related to the decrease of the three genera in the HR group. Therefore, we inferred that the HR diet changed the concentration or activity of the fermentation products and metabolites in the hindgut. However, what role these genera played and how they were associated with the intestine need to be clarified.

## 5. Conclusions

The present study revealed a detailed picture of the colonic chyme-associated microbiota after an HR diet, showing it could reduce the bacterial richness and diversity in the colon of goats. Although the slaughtering characteristic and color of the meat in goats has improved by feeding the HR diet over a short time, it may inhibit the growth of intestinal bacterial communities. Colonic concentrations of SCFA increased and pH decreased with the HR diet, which could increase the health risk to growing goats.

## Figures and Tables

**Table 1:** The meat quality of growing goats fed the control and high-rice diet ^a^.

Items		Con	HR	SEM	*p*-Value
pH					
	pH, 45 min	6.50 ± 0.30	6.33 ± 0.04	0.06	0.251
	pH, 24 h	5.56 ± 0.03	5.45 ± 0.14	0.03	0.110
	ΔpH	0.94 ± 0.31	0.88 ± 0.14	0.07	0.690
Meat color, 45 min					
	Redness (a*)	16.9 ± 2.53	17.6 ± 0.91	0.53	0.562
	Yellowness (b*)	4.28 ± 0.22	2.81 ± 0.82	0.28	0.006
	Lightness (L*)	48.7 ± 2.84	41.3 ± 2.75	1.36	0.001
Meat color, 24 h					
	Redness (a*)	19.4 ± 2.87	19.8 ± 0.62	0.57	0.757
	Yellowness (b*)	8.51 ± 1.01	7.28 ± 1.14	0.35	0.076
	Lightness (L*)	49.4 ± 2.36	42.6 ± 2.13	1.20	<0.001

^a^ Con: control diet; HR: high-rice diet.

**Table 2:** Spearman’s correlation analysis between colonic bacterial community and metabolites in growing goats ^a^.

Items	pH	Acetate	Propionate	Total VFA	LPS	ALT	AST	LACT	ALP	AMY
*Eubacterium_coprostanoligenes_group*	−0.64 *	0.31	0.52	0.31	0.23	0.50	0.37	−0.63 *	0.17	0.41
*Christensenellaceae_R-7_group*	0.61 *	−0.65 *	−0.59 *	−0.65 *	−0.65 *	−0.62 *	−0.80 **	0.45	−0.31	−0.63 *
*Oscillibacter*	−0.56	0.47	0.38	0.47	0.33	0.63 *	0.70 *	−0.55	0.44	0.72 **
*Family_XIII_AD3011_group*	0.50	−0.71 **	−0.60 *	−0.71 **	−0.49	−0.52	−0.81 **	0.37	−0.43	−0.70 *
*Phocaeicola*	0.71 **	−0.53	−0.45	−0.53	−0.70 *	−0.64 *	−0.82 **	0.71 *	−0.65 *	−0.93 **
*Prevotellaceae_UCG-004*	0.71 **	−0.75 **	−0.80 **	−0.75 **	−0.76 **	−0.54	−0.78 **	0.40	−0.54	−0.63 *

^a^ (*) 0.01 < *p <* 0.05; (**) *p <* 0.01.

## Data Availability

The data presented in this study are available on request from the corresponding author. The availability of the data is restricted to investigators based in academic institutions.

## Supplementary Materials

The following are available online at https://www.mdpi.com/article/10.3390/ani11082306/s1, Table S1: Ingredients and nutrient levels of the experimental diets (air-dried basis), Figure S1: Rarefaction curve for each sample (Con1-Con6, HR1-HR6 are colonic samples of goats fed with 55% or 90% concentrate, separately).
