# Supplementary material for: Microbiome-Metabolites Analysis Reveals Unhealthy Alterations in the Gut Microbiota but Improved Meat Quality with a High-Rice Diet Challenge in a Small Ruminant Model
Source: Animals (Basel). 2021 Aug 5;11(8):2306. doi: 10.3390/ani11082306 (PMC8388442; doi:10.3390/ani11082306)
Supplement: Supplementary file 1 [file animals-11-02306-s001.zip › animals-1254265-supplementary.pdf]

Table S1. Ingredients and nutrient levels of the experimental diets (air-dried basis) [a]

Item	Con	HR
Rice straw	45.0	10.0
Rice with shell	33.2	54.3
Soybean meal	9.60	15.7
Wheat bran	6.00	9.80
Fat powder	3.20	5.20
Calcium carbonate	0.50	0.80
Calcium bicarbonate	1.10	1.80
Sodium chloride	0.60	1.00
Premix[b]	1.00	1.40
Nutrient levels4, % of DM (dry matter)		
Crude protein	13.5	17.6
Crude ash	9.34	9.12
Crude fat	4.18	6.01
Neutral detergent	49.8	38.4
Acid detergent fiber	36.5	9.51

[a]Con: control diet; HR: high rice diet; [b]Premix composition per kg diet: 68 mg FeSO4·H2O, 44 mg CuSO4·5H2O, 411 μg CoCl2·6H2O, 1.70 mg KIO3, 211 mg MnSO4·H2O, 126 mg ZnSO4·H2O, 56 μg Na2SeO3, 462 mg MgSO4·7H2O, 737 IU vitamin A, 8.29 mg vitamin E, 5.1 g carrier zeolite powder; 4Nutrient levels were measured values.

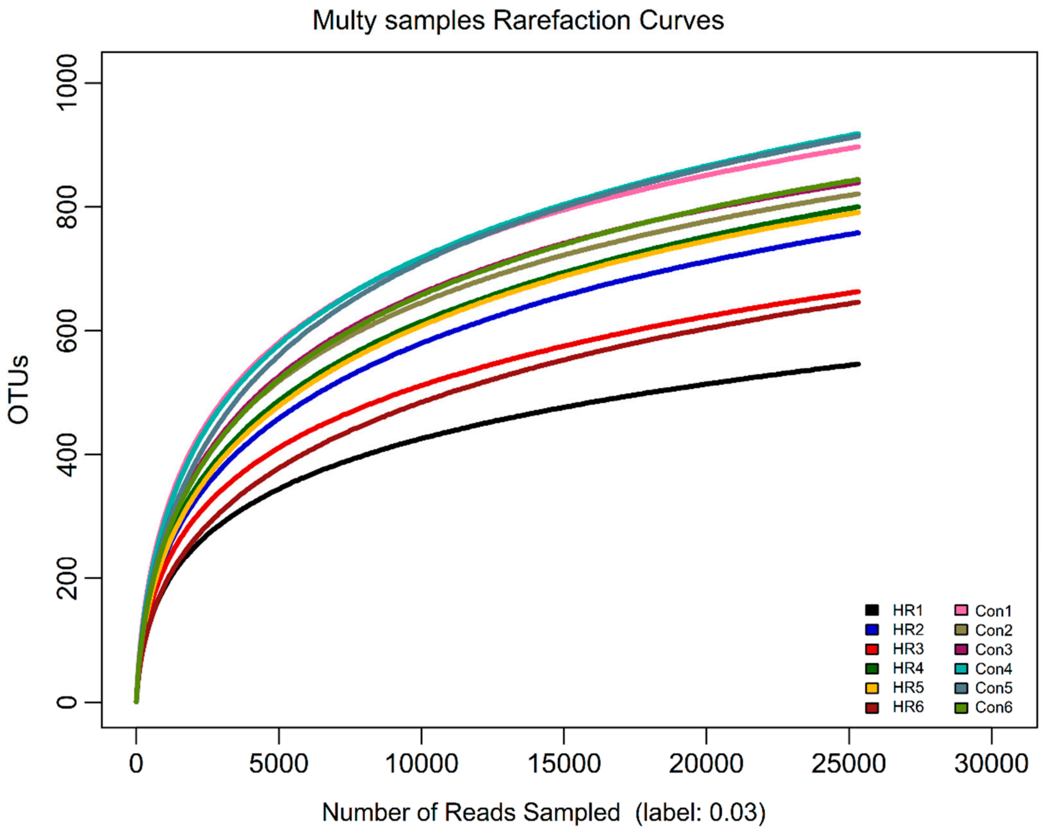

Figure S1: Rarefaction curve for each sample (Con1-Con6, HR1-HR6 are colonic samples of goats fed with 55% or 90% concentrate, separately).
